# Epidemiological characteristics and genotype distribution of low-risk human papillomavirus infection among women in Jingzhou, Central China: a cross-sectional study

**DOI:** 10.1186/s12879-026-13885-4

**Published:** 2026-06-25

**Authors:** Shangjie Zheng, Xiaoxue Wu, Wenjun Li, Xueyan Xu, Ping Gong

**Affiliations:** Department of Pathology, Jingzhou Hospital Affiliated to Yangtze University, No. 26 Chuyuan Avenue, Jingzhou District, Jingzhou, Hubei Province 434020 China

**Keywords:** Human papillomavirus, Low-risk HPV, Epidemiology, Genotype distribution, Cross-sectional study, Jingzhou, China

## Abstract

**Background:**

To systematically analyze the epidemiological characteristics and genotype distribution of low-risk human papillomavirus (LR-HPV) infection in Jingzhou, China, and to provide evidence for optimizing regional strategies for the prevention and control of HPV-related diseases.

**Methods:**

This retrospective cross-sectional study included 24,027 women who underwent cervical cancer screening and concurrent HPV genotyping at Jingzhou Hospital Affiliated to Yangtze University between October 2022 and September 2025. A flow-through hybridization technique was employed to detect 27 HPV genotypes (17 high-risk and 10 low-risk types). The prevalence and genotype distribution of LR-HPV were analyzed, with further stratification by age and season.

**Results:**

The overall HPV infection rate among the 24,027 participants was 25.94% (6,233/24,027), with a LR-HPV infection rate of 10.06% (2,416/24,027). The predominant LR-HPV genotypes were HPV81 (3.33%), HPV61 (2.55%), and HPV44 (1.63%), which differed from the detection rates of the traditionally common LR-HPV types HPV6 (0.82%) and HPV11 (0.32%). The age stratification analysis revealed a U-shaped bimodal distribution of LR-HPV prevalence, with peaks in women aged ≤ 20 years (16.02%) and over 60 years (19.15%) (*P* < 0.001). Single genotype infections predominated. Seasonally, LR-HPV prevalence was highest in autumn (10.78%) and lowest in summer (9.50%), but the differences were not statistically significant (*P* > 0.05).

**Conclusion:**

LR-HPV infection among women in Jingzhou is characterized by a unique regional genotype distribution dominated by HPV81, 61, and 44, with a bimodal age-specific prevalence pattern involving women aged ≤ 20 years and > 60 years. This genotype profile differs from those reported in other regions of China, underscoring the importance of incorporating local epidemiological data into the formulation of regional HPV-related disease prevention and screening strategies.

**Clinical trial number:**

Not applicable.

## Introduction

Human papillomavirus (HPV) is one of the most common sexually transmitted pathogens in the female genital tract [1]. Specifically, persistent infection with high-risk human papillomavirus (HR-HPV) has been definitively established as a necessary cause of cervical cancer. This clear causal relationship has directly driven the development and implementation of global cervical cancer prevention and control strategies centered on vaccination and screening [2]. In contrast, the clinical and epidemiological significance of low-risk human papillomavirus (LR-HPV) has not been fully appreciated [3]. Although LR-HPV has significantly lower oncogenic potential than HR-HPV, its public health burden should not be overlooked. The predominant genotypes, represented by HPV6 and HPV11, account for 90% of genital warts and are closely associated with low-grade cervical squamous intraepithelial lesions [4]. These conditions are not only recurrent and prone to relapse, causing significant psychological and social distress to patients, but also lead to sustained consumption of healthcare resources, resulting in a considerable economic burden [5]. Recent studies have further suggested that under specific host conditions such as immunosuppression, certain LR-HPV types may also be associated with rare epithelial dysplastic lesions [6, 7]. Moreover, as an independent marker of sexually transmitted infection, the epidemiological characteristics of LR-HPV may not completely parallel those of HR-HPV, reflecting different transmission dynamics [ 8]. These findings suggest that defining the clinical significance of LR-HPV solely from a “noncarcinogenic” perspective may underestimate its true role in the prevention and control of sexually transmitted diseases. The distribution and epidemiological characteristics of HPV genotypes are influenced by multiple factors, including ethnicity, geography, sexual behavior patterns, economic status, and vaccination policies. Currently, most epidemiological studies still focus on HR-HPV, or describe LR-HPV data as ancillary findings in overall infection studies, lacking systematic investigation into its own infection patterns and epidemiological regularities. Particularly in central China, high-quality data on the epidemiological characteristics of LR-HPV remain very limited.

As a representative densely populated area in central China, the LR-HPV prevalence in Jingzhou has not been systematically elucidated, and data from this region are crucial for completing the overall picture of HPV infection in China. Prophylactic HPV vaccines, such as the widely used bivalent, quadrivalent, and nonavalent vaccines, have been developed to prevent infection from the most common high-risk (HR) and low-risk (LR) HPV genotypes [9]. The widely used quadrivalent and nonavalent HPV vaccines primarily cover common LR-HPV types such as HPV6 and HPV11 [10]. Characterizing the distribution of locally prevalent LR-HPV genotypes may contribute to a better understanding of regional HPV epidemiology and provide supplementary information for public health planning [11, 12].

Therefore, this study utilizes large-scale population screening data to conduct the first systematic analysis of the current prevalence of LR-HPV infection among women in the Jingzhou region. It focuses on assessing infection rates, genotype distribution patterns, and associations with age and season. The findings aim to provide a scientific basis for the development of precise HPV prevention and control strategies and vaccination decisions tailored to regional epidemiological characteristics.

## Materials and methods

### Study population

This retrospective cross-sectional study consecutively enrolled 24,027 women who underwent cervical cancer screening at Jingzhou Hospital Affiliated to Yangtze University between October 2022 and September 2025.

The inclusion criteria were: (1) age ≥ 14 years with a history of sexual activity; (2) not pregnant at the time of screening; (3) no history of immunodeficiency-related diseases (e.g., HIV infection, organ transplantation); (4) no history of total hysterectomy; and (5) Each participant was included only once (first screening record). Repeated visits were excluded.

The exclusion criteria were: (1) pregnant women; (2) history of vaginal medication use or cervical surgical intervention within the previous three months; and (3) unqualified samples (e.g., insufficient cell quantity or improper preservation).

This study was approved by the Ethics Committee of Jingzhou Hospital Affiliated to Yangtze University (Approval number: 2026-030-01), and the research process strictly adhered to the ethical guidelines of the Declaration of Helsinki. Due to the use of previously collected screening data, a waiver of informed consent was granted.

### HPV genotyping test

#### Instruments and reagents

All cervical samples were genotyped using the Human Papillomavirus Nucleic Acid Genotyping Kit (flow-through fluorescence hybridization method, Tellgen Life Science and Technology Co., Ltd., Shanghai, China), strictly following the manufacturer’s Standard Operating Procedure (SOP).

This kit enables simultaneous detection of 27 HPV genotypes, including 17 high-risk types (HPV16, 18, 31, 33, 35, 39, 45, 51, 52, 56, 58, 59, 68, 26, 53, 66, 82) and 10 low-risk types (HPV6, 11, 40, 42, 43, 44, 55, 61, 81, 83).

The primary instruments used included: a biological safety cabinet (for sample processing and nucleic acid extraction), a PCR thermal cycler (ETC811, for gene amplification), and a multifunctional flow-through point array instrument (Luminex^®^ 200™, for signal reading and analysis). All instruments were routinely calibrated and maintained in accordance with laboratory quality management standards.

#### Testing procedure

Cervical exfoliated cell samples were collected by uniformly trained healthcare professionals using specialized cervical brushes, immediately placed into collection tubes containing cell preservation solution, and stored and transported under standardized conditions.

The main testing steps were as follows:

1. Nucleic Acid Extraction: Total DNA was extracted from the sample preservation solution using the nucleic acid extraction reagent provided with the kit.
2. PCR Amplification: Using the extracted DNA as a template, a multiplex PCR system with biotin-labeled primers was employed to simultaneously amplify the conserved region of the HPV L1 gene and an internal control gene (human β-globin).
3. Hybridization and Detection: The PCR amplification products were hybridized in liquid phase with fluorescently encoded microspheres coated with probes specific to the 27 HPV genotypes. After washing, Streptavidin-Phycoerythrin (SA-PE) was added for fluorescent labeling. Finally, the multifunctional flow-through point array instrument was used to read the encoding signal of each microsphere and the corresponding fluorescence intensity.

#### Result interpretation and quality control

Result interpretation strictly adhered to the threshold criteria preset by the kit. The signal value of the internal control gene had to be > 150 to ensure sample adequacy and the validity of the testing process. Under this premise, a sample was considered positive for a specific HPV genotype if the signal value for its corresponding probe was > 150. Individual samples could test positive for one or multiple HPV genotypes simultaneously.

Each testing batch included negative and positive quality controls provided with the kit, and all control results fell within the expected ranges.

### Statistical analysis

All statistical analyses were performed using IBM SPSS Statistics version 26.0, with a significance level set at α = 0.05 (two-tailed). HPV infection rates were presented as counts and percentages.

Participants were stratified into six age groups: ≤20, 21–30, 31–40, 41–50, 51–60, and > 60 years. Based on sample collection time, the year was divided into four seasons: spring (March–May), summer (June–August), autumn (September–November), and winter (December–February).

The following statistical methods were employed: (1) The Pearson’s chi-square test was used to assess trends in infection rates across age groups and seasons, with Fisher’s exact test applied when expected frequencies were less than 5; (2) The composition of multiple infections and co-infection patterns among different HPV genotypes were analyzed using descriptive statistics, and a heatmap was constructed to visually represent the strength of co-infection associations between types.

## Results

A total of 24,027 women who underwent cervical cancer screening and concurrent HPV genotyping testing at Jingzhou Hospital Affiliated to Yangtze University between October 2022 and September 2025 were finally included in this study. The age of participants ranged from 14 to 90 years, with a median age of 45 years.

### Overall, HR-HPV and LR-HPV infection rates and genotype distribution

Among the 24,027 participants, a total of 6,233 cases tested positive for HPV, yielding an overall infection rate of 25.94% (6,233/24,027). The HR-HPV infection rate was 19.42% (4,667/24,027), while the LR-HPV infection rate was 10.06% (2,416/24,027).

Regarding HR-HPV genotypes, the three most prevalent types were HPV52 (4.85%, 1,166/24,027), HPV53 (3.41%, 820/24,027), and HPV58 (2.77%, 665/24,027).

**Fig. 1:**
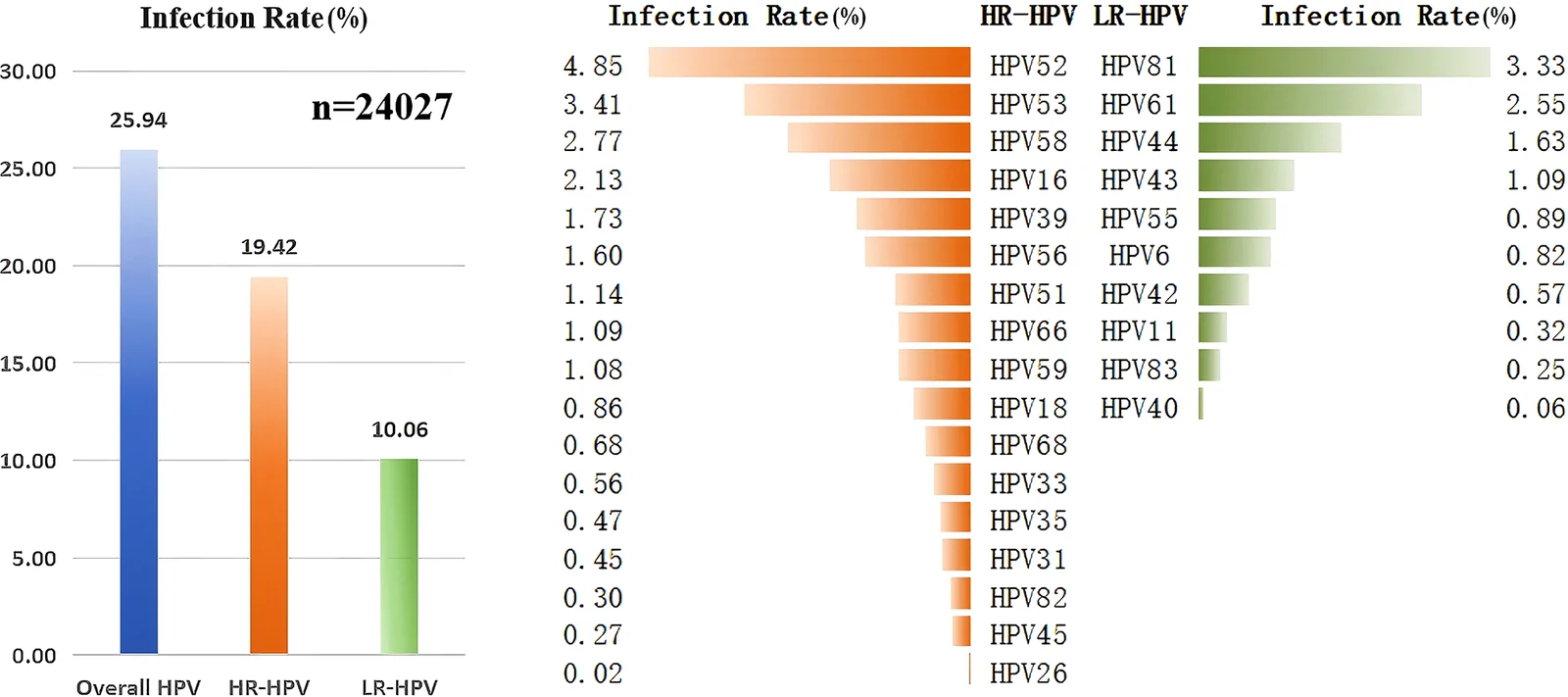
Prevalence and genotype distribution of overall HPV, HR-HPV and LR-HPV

Regarding LR-HPV genotypes, the three most prevalent types were HPV81 (3.33%, 799/24,027), HPV61 (2.55%, 612/24,027), and HPV44 (1.63%, 392/24,027). The infection rates of HPV6 (0.82%, 198/24,027) and HPV11 (0.32%, 78/24,027), which are closely associated with genital warts, were relatively low. The distribution of infection rates for each genotype is presented in Fig. 1.

### Age-stratified characteristics of LR-HPV infection

Both overall HPV and LR-HPV infection rates demonstrated significant trends with age (*P* < 0.001). The age-specific prevalence curves for overall HPV, HR-HPV and LR-HPV all exhibited a U-shaped pattern, characterized by a bimodal distribution with peaks in the ≤ 20 years and > 60 years age groups and a nadir in the 31–40 years age group. The peak overall HPV infection rate was 32.60% in the ≤ 20 years group and 41.61% in the > 60 years group, with the lowest rate of 18.18% observed in the 31–40 years group. For LR-HPV, the peak infection rate was 16.02% in the ≤ 20 years group and 19.15% in the > 60 years group, with the lowest rate of 5.76% in the 21–30 years group. For HR-HPV, the peak infection rate was 26.52% in the ≤ 20 years group and 32.77% in the > 60 years group, with the lowest rate of 14.69% in the 31–40 years group.

**Fig. 2:**
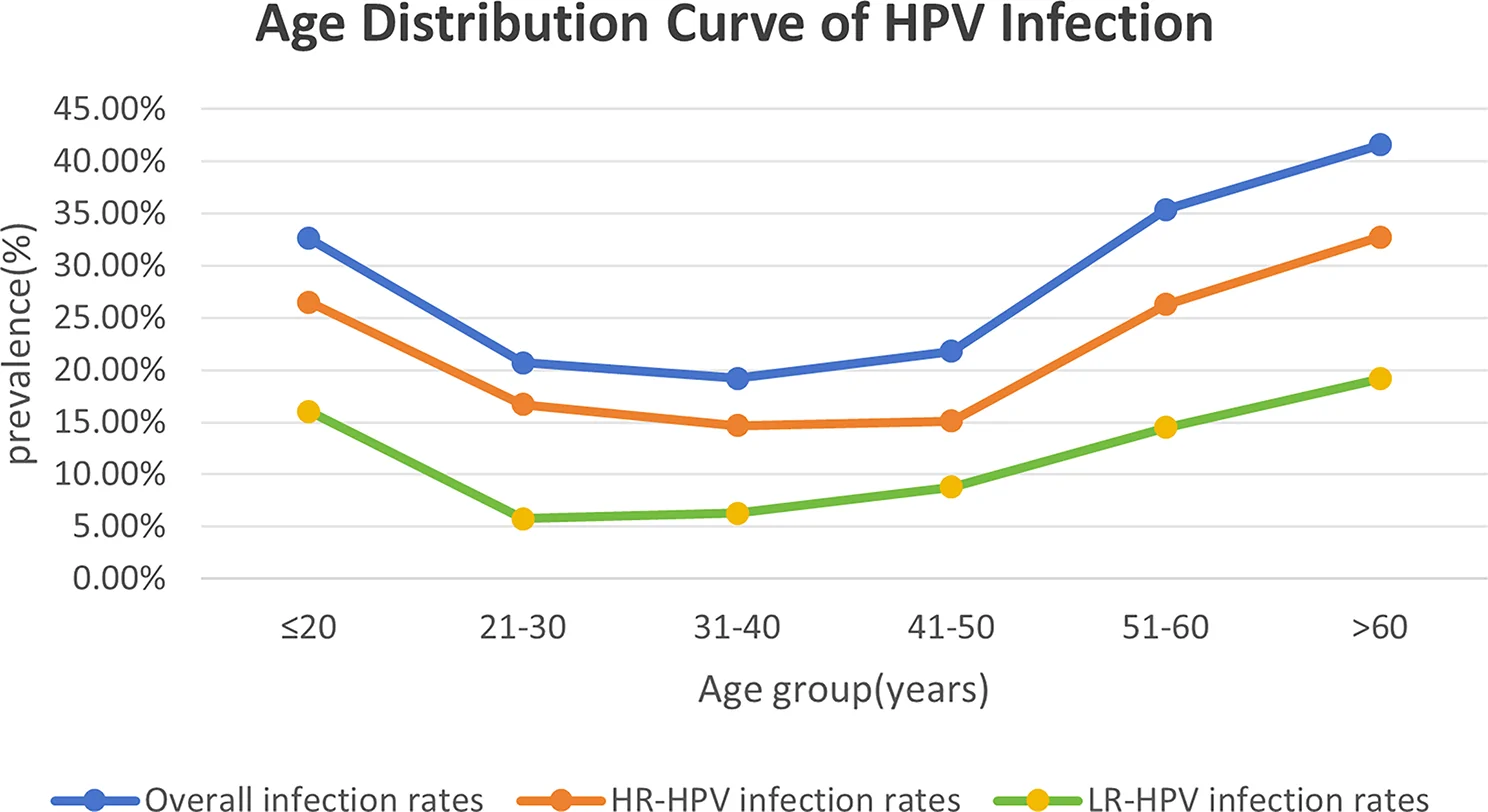
Age-specific prevalence curves for overall HPV, HR-HPV and LR-HPV

Age-stratified analysis revealed the following infection rates across age groups (≤ 20, 21–30, 31–40, 41–50, 51–60, > 60 years): overall HPV infection rates were 32.60%, 20.69%, 18.18%, 21.79%, 35.38% and 41.61%, respectively; LR-HPV infection rates were 16.02%, 5.76%, 6.29%, 8.78%, 14.51% and 19.15%, respectively; and HR-HPV infection rates were 26.52%, 16.68%, 14.69%, 15.11%, 26.27% and 32.77%, respectively. The differences in overall HPV infection rates (χ²=772.798, *P* < 0.001), HR-HPV infection rates (χ²=612.967, *P* < 0.001), and LR-HPV infection rates (χ²=477.384, *P* < 0.001) across age groups were all statistically significant. The age-specific trends in HPV (overall, HR-HPV and LR-HPV) infection rates are illustrated in Fig. 2.

### Analysis of HPV infection patterns

#### Composition of infections by risk category

Among the 6,233 HPV-positive cases, those infected exclusively with HR-HPV accounted for 61.24% (3,817/6,233), those infected exclusively with LR-HPV accounted for 25.12% (1,566/6,233), and those co-infected with both high-risk and low-risk HPV types accounted for 13.64% (850/6,233). The composition of these three infection types showed statistically significant differences across age groups (χ²=1055.065, *P* < 0.001) and across seasonal groups (χ²=20.555, *P* < 0.005).

#### Analysis of single and multiple infections

Among the 24,027 participants, single genotype infection was predominant, accounting for 19.35% (4,650/24,027). Multiple infections (≥ 2 HPV genotypes) accounted for 6.59% (1,583/24,027).

**Fig. 3:**
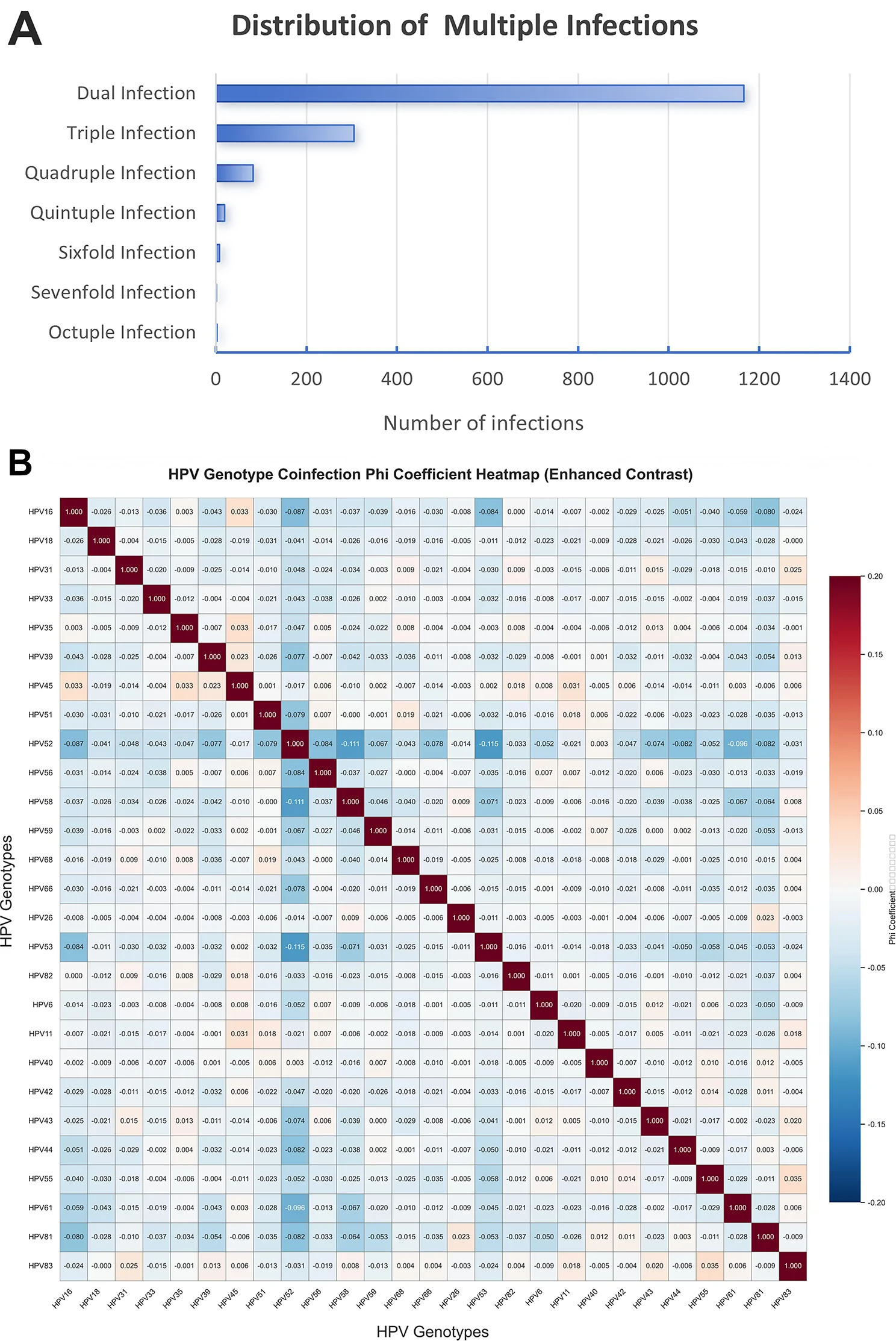
**A**. Distribution of multiple HPV infections. **B**. Heatmap of HPV genotype co-infection patterns

Among multiple infections, dual infection was the most common, comprising 4.85% (1,166/24,027) of all participants. The proportion of infections involving three to seven genotypes progressively decreased as the number of infecting types increased; however, the number of individuals infected with eight genotypes showed a slight increase compared to those infected with seven genotypes (Fig. 3A).

#### Co-infection patterns among HPV genotypes

A heatmap of genotype co-infection patterns was constructed based on co-infection analysis results from the HPV-positive population (Fig. 3B). In this heatmap, red regions indicate positive correlations (Phi > 0), suggesting a tendency for these genotypes to co-occur; blue regions indicate negative correlations (Phi < 0), suggesting potential mutual exclusivity between these types, with darker colors representing higher co-infection proportions. The diagonal represents the proportion of single-genotype infections, while off-diagonal elements represent the proportion of co-infections between two specific genotypes.

The results revealed that the most common co-infection combination was HPV16 and HPV18, accounting for 5.28% of the HPV-positive population, followed by HPV6 and HPV11, accounting for 4.64% of the HPV-positive population. Among HPV-positive individuals, co-infection rates within HR-HPV types (16/18/31/33) were substantially higher than cross-category co-infections between low-risk and high-risk types. HPV16 single infection exhibited the highest proportion (8.31%) among all genotypes.

Furthermore, the heatmap demonstrated a notable clustering trend of co-infections among LR-HPV genotypes (HPV81, HPV61, and HPV44). It may reflect shared co-infection patterns. However, causality cannot be inferred from the present cross-sectional data. The specific mechanisms need to be further validated by molecular epidemiological studies. These genotypes are precisely the main LR-HPV types prevalent in this region, and none of them are included in currently licensed HPV vaccines.

### Seasonal characteristics of HPV infection

The overall HPV infection rate showed statistically significant differences across seasons (χ²=16.013, *P* < 0.001). The highest infection rate was observed in autumn (27.57%), which was significantly higher than other seasons, while the lowest rate occurred in summer (24.43%).

**Fig. 4:**
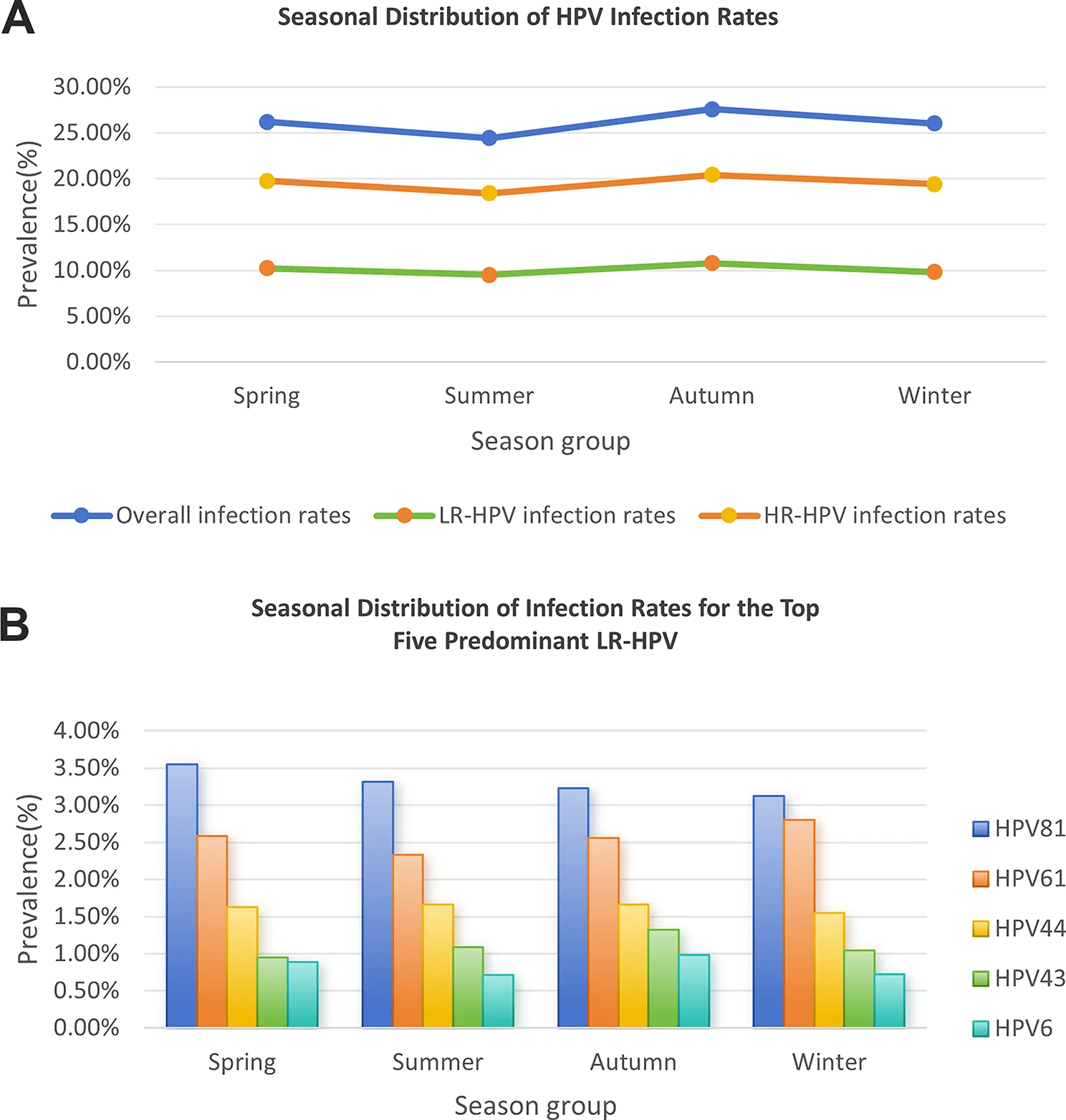
**A**. Seasonal variation in prevalence of overall HPV, HR-HPV, and LR-HPV. **B**. Seasonal variation in prevalence of the five most prevalent LR-HPV genotypes

The LR-HPV infection rate also exhibited a trend of being higher in autumn (10.78%) compared to summer (9.50%); however, this seasonal difference did not reach statistical significance (χ²=6.106, *P*>0.05) (Fig. 4A).

Regarding specific genotypes, among HR-HPV types, HPV52 maintained the highest infection rate across all four seasons: spring 5.11% (354/6,930), summer 4.51% (323/7,159), autumn 5.07% (265/5,230), and winter 4.76% (224/4,708). Among LR-HPV types, HPV81 was consistently the most prevalent genotype across all seasons, with infection rates of 3.55% (246/6,930) in spring, 3.31% (237/7,159) in summer, 3.23% (169/5,230) in autumn, and 3.12% (147/4,708) in winter.

In the seasonal stratification analysis of LR-HPV infections, the five most prevalent genotypes were consistently HPV81, HPV61, HPV44, HPV43, and HPV6 across all seasons (Fig. 4B).

### Trends in HPV infection rates over time

**Fig. 5:**
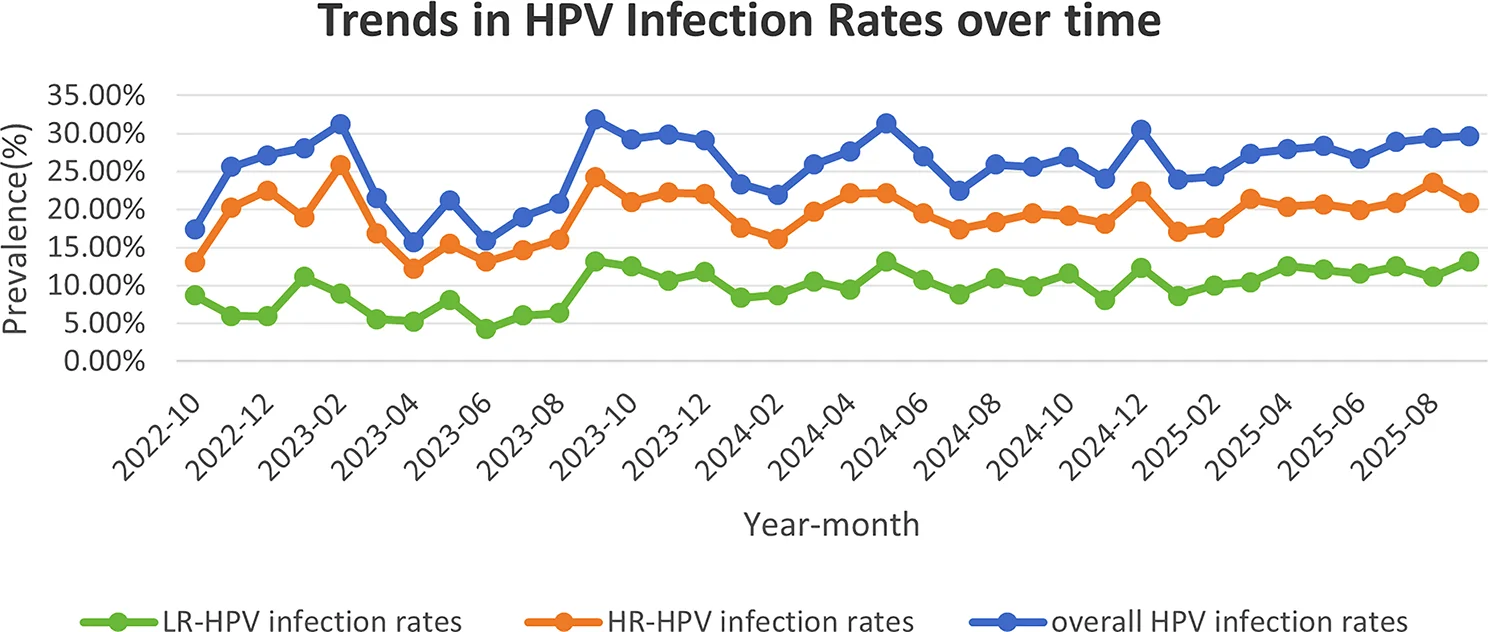
Trends in HPV infection from October 2022 to September 2025

In our study, the trends in monthly positive rates for overall HPV, HR-HPV and LR-HPV were broadly similar between October 2022 and September 2025. The overall HPV positivity rate fluctuated between 15.72% and 31.87%; the HR-HPV positivity rate showed a similar fluctuating trend (range: 12.23% to 25.88%); the LR-HPV positivity rate also varied, ranging from 4.26% to 13.20% (Fig. 5).

## Discussion

Based on a large-scale cross-sectional sample of over 24,000 women from Jingzhou in Central China, this study systematically delineates the epidemiological characteristics of LR-HPV infection in this region. Our results reveal that LR-HPV infections in Jingzhou are predominantly characterized by genotypes HPV81, HPV61, and HPV44, exhibiting notable regional specificity. Furthermore, LR-HPV prevalence displayed bimodal peaks among adolescents and middle-aged and older women, along with certain seasonal fluctuations. These findings not only fill the gap in epidemiological evidence regarding LR-HPV in this area but also could provide a regional basis for current prevention and screening strategies that primarily focus on HR-HPV types.

### Comparison of HPV genotype distribution across China and other regions

This study demonstrates that the distribution of HPV genotypes in the Jingzhou region exhibits distinct regional characteristics. With regard to HR-HPV, HPV52, HPV58 and HPV53 are the most common types in this region. This distribution pattern is consistent with the findings of previous studies in Jingzhou [13], and is similar to reports from Guangzhou [14], Changsha [15], Chongqing [16] and other regions in southern and central China, but differs somewhat from that observed in Beijing [17] and Xinjiang [18]. In particular, HPV52 and HPV58 continue to maintain high prevalence levels in central and southern China; in some areas, their infection burden even exceeds that of HPV18, second only to HPV16. This suggests significant geographical heterogeneity in the HPV epidemiological profile across China.

With regard to LR-HPV, this study found that HPV81, HPV61 and HPV44 are the predominant circulating types in the Jingzhou region. This differs markedly from the distribution pattern in some northern regions of China [19], where CP8304 predominates, but is consistent with the reported prevalence of HPV81 in the Putuo district of Shanghai [20]. Furthermore, even within the same province, marked differences can be observed between different cities. For example, the Xi’an region is dominated by HPV42, whereas Shangluo and Yan’an are dominated by HPV6 and HPV43, and HPV6 and HPV11, respectively [21]. These findings further indicate substantial regional heterogeneity in the distribution of LR-HPV genotypes across China, which has not been fully characterized in previous studies. Additionally, different ethnic populations within the same geographical region may exhibit distinct HPV genotype distribution patterns [22].

Within East Asia, neighbouring countries also exhibit distinct epidemiological characteristics. Research from South Korea reports that HPV 54 is a relatively common LR-HPV type [23], whereas a study from Japan indicates that HPV6 and HPV11—which are covered by vaccines—still account for a high proportion of cases. In terms of HR-HPV, the most common types in Malaysia, a country in Southeast Asia, are HPV 16, HPV 18 and HPV 58 [24]. These differences suggest that even within East Asia, where geographical locations and cultural backgrounds are similar, the distribution of HPV genotypes may still be influenced by a variety of factors, including population structure, behavioural patterns, surveillance systems and vaccination policies, thereby giving rise to epidemiological profiles with distinct local characteristics.

Compared with Asia, the HPV epidemiological profile in regions such as Europe and the Americas exhibits distinct characteristics. Studies in North America and Europe generally report that high-risk types such as HPV16, HPV18 and HPV45 are predominant [25–27], and the composition of LR-HPV types also differs from that in East Asia. A recent study in Greece focusing on women similarly indicated that the predominant high-risk types in that region are HPV16, HPV31 and HPV18 [28]. Furthermore, an Italian study observed HPV type distribution patterns in different populations that differed from those in this study [29]. These cross-regional comparisons further demonstrate that the epidemiological characteristics of HPV infection exhibit significant geographical variation; therefore, the establishment of epidemiological databases based on local surveillance data is of great significance for accurately assessing the disease burden and formulating prevention and screening strategies tailored to regional characteristics.

Previous studies in the Jingzhou region have primarily focused on the epidemiological characteristics of HR-HPV, while systematic analyses of LR-HPV have been relatively limited. This study found that the most prevalent LR-HPV types in this region (HPV81, HPV61 and HPV44) are not covered by current mainstream HPV vaccines. This finding may reflect differences in regional HPV genotype prevalence patterns. Alternatively, it may also be influenced by the genotype spectrum included in currently available vaccines. However, as this study did not collect individual vaccination histories, it is not possible to further assess the specific impact of vaccination on the current distribution of LR-HPV types.

### Age-related bimodal distribution of LR-HPV infection

This study observed a U-shaped age distribution of low-risk HPV infection among women in Jingzhou, with a bimodal distribution in the age groups ≤ 20 years and ≥ 60 years. This pattern is consistent with reports from other regions in China (such as Yueyang and Zhuhai) [30]. The peak in infection rates among older women is a phenomenon worthy of attention; existing literature suggests that this may be associated with various factors, including the reactivation of latent viruses, new infections, or age-related changes in the immune system [31, 32]. Elucidating the exact mechanisms will require future longitudinal studies combining behavioural and immunological indicators; this cross-sectional study provides important regional baseline data for such research.

### Seasonal variation in HPV infection

This study observed fluctuations in the prevalence of LR-HPV infection across seasons, with a slightly higher rate in autumn (10.78%) and a lower rate in summer (9.50%). However, this difference was not statistically significant (*P* > 0.05). Therefore, regarding seasonal patterns of LR-HPV infection, this study does not provide statistically robust conclusions.

Notwithstanding, the potential seasonal pattern of HPV infection remains an epidemiological question of interest. Existing literature suggests that HPV infection rates may be influenced by a combination of complex factors, including meteorological conditions (e.g., temperature, humidity) and seasonal variations in social behavior, which could jointly affect viral transmission efficiency or host immune status [33–35]. Reports of inconsistent seasonal peaks (e.g., autumn, winter, or spring) from different geographical regions further indicate the diversity and complexity of the underlying driving mechanisms [24, 36].

In conclusion, the non-significant seasonal fluctuation in LR-HPV infection prevalence observed in this study should be considered an exploratory finding. Whether it represents a true biological trend requires verification through future, well-designed longitudinal studies with adequate statistical power.

### Implications for public health strategies and study limitations

The findings of this study suggest that greater attention should be directed toward the epidemiological characteristics of LR-HPV infection in Jingzhou and other regions with similar prevalence profiles. With regard to vaccination strategies, the relationship between the locally prevalent LR-HPV genotype spectrum and the genotype coverage of currently available vaccines warrants further investigation in future epidemiological studies. In terms of screening strategies, integrating LR-HPV genotyping results into comprehensive cervical disease risk assessment systems, with particular focus on middle-aged and older women, may help compensate for previous screening inadequacies. At the health management level, optimizing the allocation of screening and health education resources based on seasonal variation characteristics could potentially enhance early detection rates of HPV-related diseases.

Nevertheless, this study has certain limitations. This single-center cross-sectional study sourced samples from a hospital pathology department, carrying a risk of selection bias. Future multi-center stratified sampling is recommended to improve representativeness. Furthermore, the lack of individual-level information on sexual behavior characteristics, vaccination history, and socio-behavioral factors limited our ability to conduct in-depth analyses of infection drivers and may have influenced the interpretation of age distribution patterns. Additionally, the cross-sectional design cannot distinguish transient from persistent infection. In addition, this study did not evaluate the associations between LR-HPV infection and cervical cytological or histopathological abnormalities, which should be explored in future longitudinal studies.

## Data Availability

This study was approved by the Ethics Committee of Jingzhou Hospital Affiliated to Yangtze University (Approval No.: 2026-030-01), and the research process strictly adhered to the ethical guidelines of the Declaration of Helsinki. Due to the use of previously collected screening data, a waiver of informed consent was granted.

## Abbreviations

HPV: Human Papillomavirus
HR-HPV: High-Risk HPV
LR-HPV: Low-Risk HPV
SOP: Standard Operating Procedure
SA-PE: Streptavidin-Phycoerythrin

## References

[1] Bray F, Ferlay J, Soerjomataram I, Siegel RL, Torre LA, Jemal A. Global cancer statistics 2018: GLOBOCAN estimates of incidence and mortality worldwide for 36 cancers in 185 countries. CA Cancer J Clin. 2018;68(6):394–424. 30207593 10.3322/caac.21492

[2] Walboomers JM, Jacobs MV, Manos MM, Bosch FX, Kummer JA, Shah KV, Snijders PJ, Peto J, Meijer CJ, Muñoz N. Human papillomavirus is a necessary cause of invasive cervical cancer worldwide. J Pathol. 1999;189(1):12–9. 10451482 10.1002/(SICI)1096-9896(199909)189:1<12::AID-PATH431>3.0.CO;2-F

[3] Arbyn M, Weiderpass E, Bruni L, de Sanjosé S, Saraiya M, Ferlay J, Bray F. Estimates of incidence and mortality of cervical cancer in 2018: a worldwide analysis. Lancet Glob Health. 2020;8(2):e191–203. 31812369 10.1016/S2214-109X(19)30482-6 PMC7025157

[4] Nielsen A, Iftner T, Nørgaard M, Munk C, Junge J, Kjaer SK. The importance of low-risk HPV infection for the risk of abnormal cervical cytology/histology in more than 40 000 Danish women. Sex Transm Infect. 2012;88(8):627–32. 22773328 10.1136/sextrans-2011-050307

[5] Wolf J, Kist LF, Pereira SB, Quessada MA, Petek H, Pille A, Maccari JG, Mutlaq MP, Nasi LA. Human papillomavirus infection: Epidemiology, biology, host interactions, cancer development, prevention, and therapeutics. Rev Med Virol. 2024;34(3):e2537. 38666757 10.1002/rmv.2537

[6] Mlynarczyk-Bonikowska B, Rudnicka L. HPV infections—classification, pathogenesis, and potential new therapies. Int J Mol Sci. 2024;25(14). 10.3390/ijms25147616 PMC11277246 39062859

[7] Sun Y, Smith C, Murray J, Zhu J, Pallavajjala A, Liang S, Klausner M, Tsai YC, Hung CF, Wu TC, et al. Comparative Analysis Reveals Recurrent Molecular Alterations in Low-Risk Human Papillomavirus 6 and Human Papillomavirus 11-Associated Squamous Cell Carcinoma of the Uterine Cervix and Vulva. Mod Pathol. 2025;38(12):100909. 41077352 10.1016/j.modpat.2025.100909 PMC12633788

[8] Chen M, Wang Y, Dong B, Zhang Y, Feng L, Zou H, Li Y, Huang Y, Huang G, Mao X, et al. Epidemiological analysis of human papillomavirus infection in senior women: a hospital-based retrospective study. BMC Infect Dis. 2025;25(1):1378. 41120924 10.1186/s12879-025-10836-3 PMC12542516

[9] Williamson AL. Recent developments in human papillomavirus (HPV) vaccinology. Viruses. 2023;15(7). 10.3390/v15071440 PMC10384715 37515128

[10] Paz-Zulueta M, Álvarez-Paredes L, Rodríguez Díaz JC, Parás-Bravo P, Andrada Becerra ME, Rodríguez Ingelmo JM, Ruiz García MM, Portilla J, Santibañez M. Prevalence of high-risk HPV genotypes, categorised by their quadrivalent and nine-valent HPV vaccination coverage, and the genotype association with high-grade lesions. BMC Cancer. 2018;18(1):112. 29382323 10.1186/s12885-018-4033-2 PMC5791190

[11] Gholamzad A, Khakpour N, Hashemi M, Gholamzad M. Prevalence of high and low risk HPV genotypes among vaccinated and non-vaccinated people in Tehran. Virol J. 2024;21(1):9. 38183101 10.1186/s12985-023-02270-1 PMC10768147

[12] Rasheed FA, Yakasai IA, Abdurrahman A, Usman A, Yusuf N. Human papillomavirus serotypes and determinants among women with invasive cervical cancer in Katsina state, Northwest-Nigeria: a multicentre study. Ecancermedicalscience. 2024;18:1714. 39021557 10.3332/ecancer.2024.1714 PMC11254414

[13] Liu S, Mei B, Ouyang Y, Li C. Prevalence and genotype distribution of human papillomavirus infection among women in Jingzhou, China: a population-based study of 51,720 women. Virol J. 2023;20(1):297. 38102627 10.1186/s12985-023-02262-1 PMC10722767

[14] Yang X, Li Y, Tang Y, Li Z, Wang S, Luo X, He T, Yin A, Luo M. Cervical HPV infection in Guangzhou, China: an epidemiological study of 198,111 women from 2015 to 2021. Emerg Microbes Infect. 2023;12(1):e2176009. 36744409 10.1080/22221751.2023.2176009 PMC9936994

[15] Xiao Y, Liu R, Wang S, Wang Y, Miao W, Chen M, Liu X, Chen Y, Wen Y, Deng Z, et al. Predicting the risk of high-grade precancerous cervical lesions based on high-risk HPV typing in Changsha China. BMC Womens Health. 2025;25(1):28. 39833893 10.1186/s12905-025-03562-0 PMC11749071

[16] Li S, He X, Li S, Su Y, Wang X, Li C. The prevalence of HPV in Chongqing, China from 2017 to 2022: a retrospective cohort study. Sci Rep. 2024;14(1):23973. 39397097 10.1038/s41598-024-74588-x PMC11471860

[17] Liu Y, Ang Q, Wu H, Xu J, Chen D, Zhao H, Liu H, Guo X, Gu Y, Qiu H. Prevalence of human papillomavirus genotypes and precancerous cervical lesions in a screening population in Beijing, China: analysis of results from China’s top 3 hospital, 2009–2019. Virol J. 2020;17(1):104. 32660490 10.1186/s12985-020-01383-1 PMC7359485

[18] Wang J, Tang D, Wang J, Zhang Z, Chen Y, Wang K, Zhang X, Ma C. Genotype distribution and prevalence of human papillomavirus among women with cervical cytological abnormalities in Xinjiang, China. Hum Vaccin Immunother. 2019;15(7–8):1889–96. 30735478 10.1080/21645515.2019.1578598 PMC6746534

[19] Wei H, Wang N, Zhang Y, Zhang J, Wang S, Zhang S. Distribution of various types of low-risk human papillomavirus according to cervical cytology and histology in northern Chinese women. Int J Gynaecol Obstet. 2014;126(1):28–32. 24792409 10.1016/j.ijgo.2014.01.020

[20] Luan H. Human papilloma virus infection and its associated risk for cervical lesions: a cross-sectional study in Putuo area of Shanghai, China. BMC Womens Health. 2023;23(1):28. 36658539 10.1186/s12905-023-02166-w PMC9854058

[21] Han X, Song G, Li Y, Dong Z, Yan X, Wang S, Tian H, Wu X, Li C, Huo Y. Prevalence and genotype distribution of human papillomavirus infection among women aged 30–65 years in Xi’an, China: a population-based study of 14,655 women. Hum Vaccin Immunother. 2021;17(12):5439–46. 34893010 10.1080/21645515.2021.2007709 PMC8903934

[22] Ma J, Yang M, Huang G. Human Papillomavirus Prevalence and Genotype Distribution Among Women With Cervical Cytological Abnormalities in Urumqi, Xinjiang, China. Diagn Cytopathol. 2025;53(7):315–24. 40099391 10.1002/dc.25467

[23] Ouh YT, Min KJ, Cho HW, Ki M, Oh JK, Shin SY, Hong JH, Lee JK. Prevalence of human papillomavirus genotypes and precancerous cervical lesions in a screening population in the Republic of Korea, 2014–2016. J Gynecol Oncol. 2018;29(1):e14. 29185272 10.3802/jgo.2018.29.e14 PMC5709524

[24] Chee CSM, Tan SSN, Voon PJ, Augustin Y, Krishna S, Mat Ali N, Wan Maharuddin IB, Tiong XT, Abdul Rahim NKB, Ismail AM, et al. Human papillomavirus (HPV) genotype distribution in Malaysia: A systematic review. BMC Infect Dis. 2025;25(1):1010. 40784935 10.1186/s12879-025-11441-0 PMC12335762

[25] Botha MH, Van der Merwe FH, Snyman LC, Dreyer GJ, Visser C, Dreyer G. Utility of Extended HPV Genotyping as Primary Cervical Screen in an Unscreened Population With High HIV Co-Infection Rate. J Low Genit Tract Dis. 2023;27(3):212–6. 37097217 10.1097/LGT.0000000000000743

[26] Monsonego J, Cox JT, Behrens C, Sandri M, Franco EL, Yap PS, Huh W. Prevalence of high-risk human papilloma virus genotypes and associated risk of cervical precancerous lesions in a large U.S. screening population: data from the ATHENA trial. Gynecol Oncol. 2015;137(1):47–54. 25667973 10.1016/j.ygyno.2015.01.551

[27] Przybylski M, Pruski D, Wszołek K, de Mezer M, Żurawski J, Jach R, Millert-Kalińska S. Prevalence of HPV and assessing type-specific HPV testing in cervical high-grade squamous intraepithelial lesions in Poland. Pathogens. 2023;12(2). 10.3390/pathogens12020350 PMC9963087 36839622

[28] Tsakogiannis D, Zografos E, Tzioga L, Zografos CG, Zagouri F, Bletsa G. Prevalence and genotype distribution of high-risk HPV genotypes among women in Greece: a retrospective analysis of 3500 women. Cancers (Basel). 2025;17(8). 10.3390/cancers17081267 PMC12026139 40282443

[29] Tartaglia E, Falasca K, Vecchiet J, Sabusco GP, Picciano G, Di Marco R, Ucciferri C. Prevalence of HPV infection among HIV-positive and HIV-negative women in Central/Eastern Italy: Strategies of prevention. Oncol Lett. 2017;14(6):7629–35. 29344211 10.3892/ol.2017.7140 PMC5755221

[30] Hou J, Zeng M, Liu C, Xie B, Li Y, Wu L, Zhu L, Li M, Zhang Z, Zhang X, et al. Cervical HPV infection in Yueyang, China: a cross-sectional study of 125,604 women from 2019 to 2022. Front Public Health. 2023;11:1210253. 37601194 10.3389/fpubh.2023.1210253 PMC10435747

[31] Li N, He L, Zhang H, Turson G, Lou J, Cheng H. Human papillomavirus prevalence, genotype distribution, risk factors, and cervical pathology association in women aged 50 years and older: a retrospective cross-sectional study in Xinjiang, China. Front Oncol. 2025;15:1694755. 41602434 10.3389/fonc.2025.1694755 PMC12832329

[32] Achdiat PA, Atiyah D, Yulianti F, Sutedja E, Gondokaryono SP, Usman HA, Maharani RH. Anogenital Warts in Geriatrics: Immunosenescence and New Sexual Contacts? A Case Report. Clin Cosmet Investig Dermatol. 2024;17:2045–50. 39282250 10.2147/CCID.S478391 PMC11397319

[33] Bruni L, Albero G, Rowley J, Alemany L, Arbyn M, Giuliano AR, Markowitz LE, Broutet N, Taylor M. Global and regional estimates of genital human papillomavirus prevalence among men: a systematic review and meta-analysis. Lancet Glob Health. 2023;11(9):e1345–62. 37591583 10.1016/S2214-109X(23)00305-4 PMC10447222

[34] Khalili SM, Rafiei EH, Havaei M, Alizadeh L, Ghahremani F, Keshavarz Z, Montazeri A, Riazi H. Relationship between human papillomavirus and serum vitamin D levels: a systematic review. BMC Infect Dis. 2024;24(1):80. 38216875 10.1186/s12879-024-09006-8 PMC10787408

[35] Wei F, Georges D, Man I, Baussano I, Clifford GM. Causal attribution of human papillomavirus genotypes to invasive cervical cancer worldwide: a systematic analysis of the global literature. Lancet. 2024;404(10451):435–44. 39097395 10.1016/S0140-6736(24)01097-3

[36] Dickey BL, Coghill AE, Ellsworth GB, Wilkin TJ, Villa LL, Giuliano AR. An Updated Systematic Review of Human Papillomavirus Genotype Distribution by Cervical Disease Grade in Women Living With Human Immunodeficiency Virus Highlights Limited Findings From Latin America. Sex Transm Dis. 2021;48(12):e248–54. 34110738 10.1097/OLQ.0000000000001412 PMC8525704

